# Women workers’ rights and managers’ point of view: a qualitative approach to maternity leave and breastfeeding

**DOI:** 10.1590/0102-311XEN059025

**Published:** 2026-07-06

**Authors:** Marília Neves Santos, Alanna Santos de Oliveira, Bruna Souza Pereira Fagundes, Ester William Ferreira, Gabriela Buccini, Leandro Alves Pereira, Luciana Saraiva da Silva, Vivian Mara Gonçalves de Oliveira Azevedo, Ana Elisa Madalena Rinaldi

**Affiliations:** 1 Universidade Federal de Uberlândia, Uberlândia, Brasil.; 2 University of Nevada, Las Vegas, U.S.A.

**Keywords:** Exclusive Breastfeeding, Working Woman, Maternity Leave, Aleitamento Materno Exclusivo, Trabalhadora, Licença-Maternidade, Lactancia Materna Exclusiva, Trabajadora, Licencia por Maternidad

## Abstract

This study aimed to learn managers’ points of view on maternity leave and breastfeeding and find promotion, protection, and support strategies for breastfeeding in the work environment. This qualitative study, using Laurence Bardin’s content analysis, was conducted with eight managers in 2023 in Uberlândia, Minas Gerais State, Brazil. We used a semi-structured script with questions about how managers organized women workers’ activities before they took their maternity leaves and after their return to work and questions about managers’ points of view about breastfeeding and maternity leave. Answers were analyzed using NVivo. We established three big topics from the content of the interviews, namely: (1) managers’ point of view regarding the duration of maternity leave and breastfeeding; (2) rights, incentives, and work conditions; and (3) the organization of the work environment during maternity leave. We verified compliance with the 4-month maternity leave provided for in the Brazilian Consolidation of Labor Laws, highlighting the disagreement of leave duration and the health recommendation for exclusive breastfeeding of up to six months. We also found that managers’ lack of awareness about government programs encourage companies to support breastfeeding by tax reduction benefits and extended maternity leaves. Managers’ point of view and personal experiences permeate knowledge about laws protecting female workers. The pro-breastfeeding strategy only involved complying with the maternity leave duration provided by law, which indicates the need for greater investments in awareness-raising actions for managers regarding the review of the concept of maternal protection.

## Resumo

Este estudo buscou conhecer os pontos de vista dos gestores sobre licença-maternidade e amamentação e identificar estratégias de promoção, proteção e apoio para o amamentação no ambiente de trabalho. Trata-se de um estudo qualitativo, com método de análise de conteúdo proposto por Laurence Bardin, foi realizado com oito gestores em 2023 em Uberlândia, Minas Gerais, Brasil. Usamos um roteiro semiestruturado com perguntas sobre como os gestores organizavam as atividades das mulheres antes da licença-maternidade e depois que retornavam ao trabalho e sobre os pontos de vista dos gestores acerca do amamentação e da licença-maternidade. As respostas coletadas foram analisadas usando o software NVivo. Estabelecemos três grandes temas a partir do conteúdo das entrevistas: (1) o ponto de vista do gestor sobre a duração da licença-maternidade e da amamentação; (2) direitos, incentivos e condições de trabalho e (3) a organização do ambiente de trabalho durante a licença-maternidade. Verificamos o cumprimento da licença-maternidade de quatro meses, conforme previsto na Consolidação das Leis do Trabalho, destacando o desacordo entre a duração da licença e a recomendações de saúde para a amamentação exclusiva de até seis meses. Também verificamos a falta de sensibilização dos gestores sobre programas governamentais que incentivam empresas a apoiar a amamentação por meio de benefícios de redução de impostos e licenças maternidade estendidas. O ponto de vista dos gestores e sua experiência pessoal permeiam o seu conhecimento sobre as leis que protegem as trabalhadoras. A estratégia pró-amamentação envolveram apenas o cumprimento da duração da licença-maternidade prevista por lei, o que indica a necessidade de maiores investimentos em ações de sensibilização para gestores sobre a revisão do conceito de proteção à maternidade.

## Resumen

El objetivo del estudio fue conocer la opinión de los gestores sobre la baja por maternidad y la lactancia materna e identificar estrategias de promoción, protección y apoyo a la lactancia materna en el entorno laboral. Este estudio cualitativo, que empleó el método de análisis de contenido propuesto por Laurence Bardin, se llevó a cabo con ocho gestores en 2023 en Uberlândia, Minas Gerais, Brasil. Se utilizó un guion semiestructurado con preguntas sobre cómo los gestores organizaban las actividades de las trabajadoras antes de la baja por maternidad y después de su reincorporación al trabajo, así como preguntas sobre la opinión de los gestores acerca de la lactancia materna y la baja por maternidad. Las respuestas recopiladas se analizaron utilizando el software NVivo. A partir del contenido de las entrevistas, se establecieron tres grandes temas, a saber: (1) el punto de vista del gestor sobre la duración del permiso por maternidad y la lactancia materna; (2) los derechos, los incentivos y las condiciones de trabajo; y (3) la organización del entorno laboral durante el permiso por maternidad. Verificamos el cumplimiento de los cuatro meses de licencia por maternidad, tal y como se establece en la Consolidación de las Leyes Laborales de Brasil, destacando el desacuerdo entre la duración de la licencia y la recomendación sanitaria de lactancia materna exclusiva hasta los seis meses. También verificamos la falta de conocimiento de los gestores sobre los programas gubernamentales que promueven el apoyo de las empresas a la lactancia materna mediante beneficios fiscales y la ampliación del permiso por maternidad. El punto de vista de los gestores se basa en el conocimiento de las leyes que protegen a las trabajadoras, combinado con su experiencia personal. La estrategia a favor de la lactancia materna se redujo al cumplimiento de la duración del permiso por maternidad prevista legalmente, lo que indica la necesidad de realizar mayores inversiones en acciones de sensibilización de los gestores con respecto a la revisión del concepto de protección materna.

## Introduction

Women face higher unemployment rates, lower salaries, difficulties in professional growth, and greater informality despite their growing insertion in the labor market ^1^. In Brazil, from the fourth quarter of 2012 to the fourth quarter of 2017, the number of women working for themselves increased by 18.6% ^2^. The COVID-19 pandemic worsened this situation. The period saw an increase in the number of self-employed women, so-called entrepreneurs who actually fought to survive in the face of precariousness and uncertainty ^3^.

Actions to protect breastfeeding aim to guarantee, through legislation, the women’s right to feed their children and protect them against the unethical marketing of products that compete with this practice ^4^. Guaranteed paid maternity leave for women in the formal labor market constitutes one of the preconditions for the right to care and be cared for ^5^. Pregnant and breastfeeding workers need adequate time to give birth, recover from childbirth, and breastfeed their children and protection to ensure that they remain in their jobs due to pregnancy or maternity leave ^6^.

The *Brazilian Federal Constitution*, Art. 7, VXIII, guarantees pregnant employees 120 consecutive days of leave without harm to their employment or payment, which may begin on the first day of the ninth month of pregnancy, unless brought forward by medical prescription ^7^. *Decree n. 6,690/2008* regulates the extension of maternity leave for an additional 60 days, provided for in *Law n. 11,770/2008*, for female employees assigned to bodies and entities that are part of the direct, autonomous or foundational Federal Public Administration. Companies registered in the Brazilian Corporate Citizen Program [*Programa Empresa Cidadã*] can extend maternity leaves for their female employees from 120 to 180 days and increase paternity leave by 15 days in exchange for tax exemptions as a government benefit ^8^.

In addition to these rights and incentives, Brazil has created a strategic action called Working Women Who Breastfeed [*Mulher Trabalhadora que Amamenta* - MTA] to create a culture of support, protection, and respect for breastfeeding in public and private companies to promote working mothers’ and their children’s health, directly benefitting companies and society ^9^. Few studies in the literature have evaluated the adoption and implementation of breastfeeding support rooms in Brazil; one study has assessed the southern states of the country, finding challenges regarding the standardization of internal processes for maintaining rooms, their infrequent use, and difficulty in keeping tutors trained by the Brazilian Ministry of Health ^10^.

Despite legal protection of maternity leaves, the workplace shows inadequate and insufficient support so women can carry out their activities and continue breastfeeding ^11^. Breastfeeding remains a caregiver archetype ^12^, in which women configure the sole responsible and decision-makers regarding the activity without any perspective of conduct or knowledge from managers in the workplace ^11^. Caring in general (especially that of children) has traditionally been attributed to women, which may be related to motherhood. The predominance of women in child care can lead to identifying or associating care with the female role and making care an integral part of women’s self-concept ^13^. The success or failure of breastfeeding should not be seen solely as women’s responsibility. The support and the environment in which they live greatly influence their ability to breastfeed. Governments and society have a broader responsibility to support women via policies and programs in the community ^14^.

Therefore, understanding managers’ point of view regarding work conditions, laws, and policies is essential to find the changes Brazil requires to consistently support working mothers to continue breastfeeding after returning to work. Managers have a critical role in women’s breastfeeding success. Thus, they should learn breastfeeding benefits ^15^ and create favorable actions for breastfeeding maintenance in their companies ^10^. This study aims to understand managers’ points of view on maternity leave and breastfeeding and find the presence of promotion, protection, and support strategies for breastfeeding in the workplace.

## Methods

In this qualitative study, data were collected from January to August 2023, in Uberlândia, Minas Gerais State, Brazil. This study derives from a PhD dissertation in which we interviewed paid female workers and invited their managers to participate in this research via emails and/or phone calls. We invited managers and described the objectives and reasons for this research to them. Managers consisted of professionals in the human resources (HR) departments and company owners (in the case of small and/or family businesses). The interviewed managers were contacted to participate in this study after interviews were conducted with formally employed women working at the same location. The varying contexts reflect the sample of these women, who were selected from the 2019 Annual Social Information Report [*Relação Anual de Informações Sociais*] database. Women working in formal employment are mainly specialized in the education and health sectors.

Sample size was determined by thematic saturation once no new information that was relevant to the objectives of this study was found. All institutions that agreed to participate in this research were included in this study, which brought quite heterogeneous contexts, from the perspective of the economic sector (education, health, food, finance) and from the legal framework of employment relationships - Brazilian Consolidation of Labor Laws (CLT, acronym in Portuguese) in private companies and the Unified Legal Regime in public universities.

Managers’ participated in online interviews via video calls in which the interviewer (M.N.S.) followed a semi-structured script. The research team prepared the script, and one of the authors of this article (G.B.), who had previous experience with this type of material, revised it. This script had two main axes: (1) how companies organize the activities carried out by women during maternity leave and after her return and (2) the manager’s point of view on breastfeeding and maternity leave duration. We also collected participants’ sociodemographic data (age, gender, and education) and their work location and department.

We used Zoom (https://zoom.us) for the interviews with no time limit, according to interviewees’ availability. We interviewed each participant once as the content met our research objectives. Only the interviewer and the participant were present in the room. We recorded all interviews, which were later transcribed by a specialized company. The interviewer and first author of this study checked the transcripts for accuracy. The interviewer is a nutritionist and employee of the Human Milk Bank of Uberlândia. However, her participation in a graduate program as a PhD student was highlighted in her presentation to interviewees.

Laurence Bardin’s content analysis was used to analyze interviewees’ responses. This method is defined as a set of communication analysis techniques that uses systematic and objective procedures to describe the content of messages ^16^. Content analysis comprises three steps: pre-analysis (material organization, exhaustive observation, representativeness, homogeneity, relevance, and exclusivity); material exploration (floating reading and creation of a table of contents organized into indicators), and result processing (coding and inference, in which the data are systematically transformed and aggregated into units).

We imported the interview transcripts onto NVivo 1.7.1 (https://lumivero.com/products/nvivo/) for further reading and code or category creation. The key ideas in the statements were identified during analysis, based on which the central topics and subtopics were established (Box 1). Some statements were included in this study to exemplify the discussed topics. We used the acronym “GE” (from Portuguese *gestor*, “manager”) to identify the speeches, followed by a number to represent the interview order.

**Box 1:** Organization and description of central themes, their respective subthemes, and description based on content analysis.

TOPICS	SUBTOPICS	DESCRIPTION
Topic 1: Managers’ point of view on breastfeeding and maternity leave duration	- Personal experience with breastfeeding - Maternity leave duration - Child’s rights and importance	This topic covered managers’ personal experiences, knowledge, and opinions
Topic 2: Rights, incentives, and work conditions	- Breaks after returning from maternity leave - Breastfeeding support rooms - Brazilian Corporate Citizen Program	This topic addressed the aspects provided for in the maternity protection law mentioned by the interviewed managers
Topic 3: Organization of the company to accommodate maternity leaves	- Team sizing - Remote work - Pro-breastfeeding actions	This topic comprises the internal functioning of the company/public body during the absence and return of women workers after maternity leave

Participants signed informed consent forms. This research was approved by the Research Ethics Committee of the Federal University of Uberlândia (CAAE: 39289020.4.0000.5152).

## Results

In total, eight managers participated in this study (six women and two men), with ages ranging from 39 to 58 years. Most had a graduate degree (75%). Participants worked in the following locations: private early childhood education school (company owner); private middle and high school (HR professional); general hospital and maternity (HR professional); restaurant (company owner); municipal education department (head of human development); public university (vice-rector of HR management); assistance, study, and research foundation (HR manager); Brazilian financial institution (people manager). Of the three companies that offered 6-month maternity leaves, two were federal and municipal autonomous agencies and one was registered in the Brazilian Corporate Citizen Program. No company (represented by the participating managers) had a breastfeeding room. Note the context heterogeneity in this study and its differing organizational and legal realities, which illustrate various institutional situations, with distinct legal and budgetary frameworks.

The interviews averaged 30 minutes. Content analysis obtained three main topics: Topic 1: managers’ point of view on breastfeeding and maternity leave duration; Topic 2: rights, incentives, and work conditions; and Topic 3: organization of the company to accommodate maternity leaves (Box 1).

Companies that offered 6-month maternity leaves considered it an appropriate amount of time. Most managers of the companies that offered four months considered it an inadequate amount of time. Most interviewees were unaware of the Brazilian Corporate Citizen Program.

In Topic 1, we observed knowledge regarding labor legislation related to maternity protection. Overall, two public institutions and one private (Brazilian Corporate Citizen Program) offered 6-month maternity leaves. Managers deemed that 6-month maternity leaves were more compatible with health organizations’ recommendations for exclusive breastfeeding. However, some participants stated that the situation of the companies requires consideration and claimed the absence of incentives for doing anything differently from what is provided for in the Brazilian CLT, showing the tension between what is thought and what is possible to be done in the work environment.

“*Oh, it is very complicated because, if you think about the breastfeeding topic the way science is talking about it, you know? The ideal would be for her to keep breastfeeding for at least six months. This should be the minimal duration for the women breastfeeding*” (#GE1).

“*But it’s obvious that we also need to think from the other side of the coin, right? The other side is the companies’ side, the organizations’ side, right? They need their employee back. They need to keep their activities going on, right, including to preserve the jobs, right?*” (#GE2).

“*We know it’s short. Okay, but we see this difficulty, right? There isn’t much incentive for us to do things differently, right?*” (#GE3).

Some participants shared their personal experiences with breastfeeding and returning to work after maternity leave, including a male manager, who reported his and his wife’s experience.

“*Oh, it is essential* [6-month maternity leave]*, especially for the mother. It really is a physical and emotional issue. It’s not easy. We say it but it’s really not easy. There is a lot of discomfort, including with the body itself; and, in the beginning, it hurts a lot, right? In fact, the body is not prepared until up to four months. In this case, she* [his wife] *only breastfed. So, there are several night feeds. So, it* [6-month maternity leave] *is essential, even for us to be able to adapt to our new live and for the baby too because there is still no routine, no right time, you know, of feeding*” (#GE4).

“*The first daughter, when we talked, and we decided that the mother would stay home to take care of her, right? In that period, at least two years, right? And it’s a fact, right? When breastfeeding, we know how important it is, right? For the mother, it’s very important; for the child it’s very important. As a state policy, right, it’s very important because, as you create conditions for this to happen from the point of view of the baby’s health and from the point of view of safety, this baby will have to go to the doctor; from the mental point of view, that whole relationship with the mother, right, is essential during this period. So, I followed this closely at home*” (#GE2).

Some statements mentioned knowledge about the importance of breastfeeding, especially for children, and how this right must be guaranteed.

“*Yes, I think... well, I think so, right? In my layman’s view on the subject, I don’t have much authority to speak, but what I think is that universal phrase, right? ‘Human milk is the most complete food in the world’. So, I am super in favor of it. I think this moment between mother and child is important, and hospitals need to be trained to provide all the advice and guidance to mothers*” (#GE5).

“*Yes, it is a right that the child has*” (#GE6).

In Topic 2, managers mentioned rights, incentives, and work conditions toward women workers after they return from their maternity leaves (for example, two 30-minute breaks or the possibility of ending the work day one hour earlier) and appropriate areas for breastfeeding at work. The right to breaks during the working day was the most reported topic, although a manager reported a lack of knowledge about this right.

“*Then she returns* [from the four-month maternity leave] *and still has an hour to leave at whatever time she thinks is best to deal with breastfeeding* (...)*. By their unanimous preference, they leave* [earlier]*. I think that for personal reasons, they prefer, let’s say, to get organized at home, right? Maybe leave the prepared milk there, right, already collected. Most prefer to leave earlier*” (#GE5).

“*We... I didn’t know that, so I didn’t know there was this law or this right. Honestly, I don’t know*” (#GE3).

A company - represented by a manager - opened a breastfeeding support room at the end of 2019, which stopped working when the COVID-19 pandemic started. For one of the managers, this strategy can be implemented in his workplace.

“*Oh, currently, there are no breastfeeding rooms. It was opened shortly before the pandemic. That had an impact, right? It closed because of the pandemic and then we resumed it, there was that too, só... we assessed it wasn’t the right time to have adherence*” (#GE7).

“*I think the university needs to look at it and understand that this moment is important and necessary for women. If she returns and is still breastfeeding and doesn’t have first an adequate space and time to do it, right, the natural tendency is for her to abandon, right, this breastfeeding moment. So, I think you can present us with a proposal, and this proposal will be incorporated, right? I believe that we can do something like that, yes*” (#GE2).

The Brazilian Corporate Citizen Program is part of the policy of only one institution (represented by its manager), the same institution that had a breastfeeding support room. Of the other companies, some managers were unaware of the program but showed interest in the proposal.

“*Because the program only benefits those who are part of it: besides the tax incentive, when we increase the maternity leave, we can promote a better quality of life to employees and their families. It is a win-win strategy*” (#GE7).

“*I had never... I thought it is really nice. I think it’s something we have to think about it, have it well-thought-out, but it would be very nice to bring it here*” (#GE5).

We questioned managers about their company organization regarding the activities carried out by working mothers who take maternity leave and how the company receives them after the end of that period. Most managers stated that companies redistribute activities among other employees. Others said being able to hire new employees for temporary work.

“*Usually, when it is a technical function, this substitution doesn’t happen, right? It turns out that the team shares it, right, this person’s activities*” (#GE4).

“*We hire and select new employees, right? The person arrives knowing that it is probably a temporary contract because we don’t have... it is not a big school. So, sometimes we don’t have the opportunity to relocate this new person, so we already hire the person knowing that she won’t have a job when the mother returns to work, you know?*” (#GE8).

“*After the mother returns, we move her to a safe sector. Then, she goes on maternity leave. When she returns and is still breastfeeding, she remains in the safe sector and only returns to her job when her baby is six months old*” (#GE1).

The possibility of remote work was a reality for a few female workers who tried this format during COVID-19. After the extinction of the Federal Law that authorized remote work, some places remained in this format.

“*Look, for a long time, pregnant workers who returned from leave were doing remote work, right? But, for a while now, it depends on the unit, it depends on the manager, the type of work. So, everything is discussed and there is no guarantee that she will work remotely*” (#GE7).

“*Our pregnant women and mothers who have returned from maternity leave had a lot* [of benefits] *beyond the revocation of the Federal Law on remote work*” (#GE8).

In addition to the legal provisions, we investigated possible pro-breastfeeding actions companies may have carried out. Among the participating managers, only one reported incentive activities for breastfeeding, such as the extension of breastfeeding breaks during the working day until the child is one year old.

“*This benefit is granted by the company; these extra five or six months with breaks until completing a year in this case, right? From the mother’s return up to the child reaching one year of age* (...)*, we have a collective agreement that includes all employees, even the ones that have adopted. When she returns from leave, she must request and present a medical report that she is breastfeeding only once. She does not have to present that report every month*” (#GE7).

## Discussion

This study aimed to understand managers’ point of view regarding maternity leave duration and breastfeeding after workers’ return to work and identify the rights for maternity protection and pro-breastfeeding practices that complement legal demands. Our results contribute to understanding how companies and managers encourage working mothers to continue breastfeeding after returning to work.

All interviewed companies minimally complied with the provisions of the Brazilian CLT by offering 4-month maternity leaves to their workers (although most managers deemed that the period disagreed with health recommendations regarding exclusive breastfeeding of up to six months). In this case, the tension between the minimum compliance with legislation and the absence of organizational incentives to go beyond the law stands out. Also, participants showed no unanimous knowledge about federal programs that encourage companies to support breastfeeding by extending maternity leave from four to six months via tax reduction benefits (such as the Brazilian Corporate Citizen Program, which has been in force since 2008). These results are similar to those in another study conducted in a capital in the southern region of Brazil, in which managers acknowledge the benefits of breastfeeding but are unaware of the legislation regarding breastfeeding working women ^11^.

CLT provides for the right to breaks during the working day or a reduction in daily working hours by one hour for 60 days after returning to work ^17^, which managers considered as the main strategy to protect breastfeeding during return to work (although some interviewees were unaware of this right). This result is similar to the reports of a scoping review on the main strategies to protect breastfeeding and the potential impacts on achieving the Sustainable Development Goals ^18^.

In comparison with the International Labour Organization, the gap in the Brazilian CLT refers to it limiting the maximum duration of breaks to 60 days after workers’ return to work, whereas the international legislation provides for the right to one or more daily breaks or a reduction in working hours to feed a child ^19^. The MTA Strategy, proposed in 2015 by the Brazilian Federal Government, contributes to a change in culture within companies toward greater breastfeeding support.

Noncompliance with Brazilian Law - such as failing to guarantee breaks after returning to work - and the lack of knowledge of government action strategies such as the Brazilian Corporate Citizen Program highlight the need for an effective approach to raise awareness and support managers in creating and maintaining breastfeeding support programs at the workplace. These results highlight that the work environment still fails to greatly welcome and protect breastfeeding, reinforcing the role of women as the sole responsible persons for it. The booklet for the Breastfeeding Working Mothers, prepared by the Brazilian Ministry of Health, aims to guide female workers about their rights and maintain breastfeeding after returning to work. The Brazilian Ministry of Health also launched a guide ^20^ to help companies to install breastfeeding support rooms in the workplace in partnership with local health authorities, reinforcing the responsibility of companies in supporting breastfeeding ^9^. This initiative enables the creation of a suitable space for breastfeeding women in the workplace and promotes gender equality. Although managers reported the lack of incentives to do anything different from what is provided for in the Brazilian CLT, we must shed light to the existence of other possibilities.

A study in England ^21^ found managers’ lack of support as a major source of women’s dissatisfaction and the reason for some women staying away from work for longer than they wanted or for resigning after maternity leave. In a Brazilian study ^22^, a little more than half of the interviewed women reported having received support from coworkers and superiors to continue breastfeeding after returning to work, finding no difference in the support offered by female or male coworkers. The MTA support strategy constitutes an action to train healthcare providers to teach about the topic in their states/municipalities and raise awareness in public and private companies’ managers about the importance of maintaining breastfeeding for the health of children, women, and society ^4^. Until this moment, Uberlândia has no MTA tutors.

A study in southern Brazil ^23^ found that managers have positive supporting actions regarding breastfeeding if they understand the positive impact of these actions on women workers’ productivity. In contrast, when managers neither have knowledge regarding the law nor understand the importance of breastfeeding for female workers’ productivity and quality of life, actions to promote breastfeeding inside companies fail to materialize.

We have emphasized the importance of maternity leave for women, but little has been discussed about its benefits to the family. Considered a relatively male space, the Brazilian labor market remains a place in which women suffer all kinds of discrimination, which means they need to make extra efforts to balance productive and reproductive activities ^24^. The relationships between genders are socially produced, learned, institutionalized from childhood and transmitted throughout generation. They are historically constructed and shaped throughout the personal and social development process by behaviors that will be reproduced in adulthood ^25^. Thus, by deciding to support and favor female workers, managers outline a characteristic that favor a possible change in the culture of breastfeeding support and the presence of the mother at work ^11^.

Providing an extended maternity leave duration, and other actions to promote, protect, and support breastfeeding, can be more effective in large companies, which have greater resources ^26^. This became evident in the actions mentioned by the manager of one a company that has more than 80,000 employees throughout Brazil (of which 45% are women). This company is registered in the Brazilian Corporate Citizen Program, thus providing female workers with 6-month maternity leave and encouraging continued breastfeeding by allowing breaks until the child turns one year old.

The extension of maternity leave promoted by the program fails to change the retention of mothers in the job market or their salaries for up to five years after childbirth (in comparison with employees who had access to 120 days of leave). This positive result of the program even include the most educated female workers and in the financial sector (which tends to be more competitive) ^26^. In the state of Rio de Janeiro, *Resolution n. 2,359/2020*, proposes a breastfeeding leave at the end of the maternity leave, granting workers 90 days (divided into three periods of 30 days) upon presentation of a detailed medical report ^27^.

Due to the pandemic, a manager reported that the breastfeeding support room, which had just opened in the company headquarters in Brasília, was inoperative at the time of the research. breastfeeding rooms configure an important low-cost strategy for female workers to keep breastfeeding after returning to work. A study in southern Brazil ^10^ concluded that countries should invest in promoting breastfeeding in the work environment by creating these rooms. However, managers’ perception regarding the implementation of breastfeeding support rooms indicates a more negative than positive view of the specific and adequate space for breastfeeding, with justifications related to factors such as cost, physical space, and the functionality of this space ^11^. Although the literature is scarce on the subject, there is a belief that managers’ support of breastfeeding in the workplace is fair and can improve some organizational processes, such as trust or organizational identity ^28^.

The limitations of this study include its reduced number of interviewed managers due to low adherence to this research. We made several attempts to contact managers via email and phone calls. However, a considerable number of contacts ignored our requests. The awareness-raising work begins, including at this moment, when researchers need to convince people of the relevance of the topic, the objective of which also includes ending all discrimination against women and promote gender equality and women’s empowerment ^14^. Additionally, considering a perspective to contextualize this standpoint, we emphasize that most interviewed managers were women as we mainly interviewed people from education and health (positions generally occupied by women). Some of them had experienced breastfeeding. We know that this likely changes opinions and debates on the subject and reinforces the caregiver archetype attributed to women. A similar study mostly involving male respondents reported that they use the knowledge they acquired by personal experience as parents to meet the needs of working mothers, with 80% of the managers having children and all having been breastfed. That study recommends that healthcare providers work to raise awareness among these managers about effective ways to support working mothers ^11^.

We highlight, as a limiting aspect of our research, the heterogeneity of the participating institutions, as we know that the conditions for implementing pro-breastfeeding policies differ between public agencies and private companies. This captures a variety of perceptions and shows how the organizational environment shapes managers’ behaviors. We would probably hear different opinions if most managers only worked on the private sector, especially under no additional action to current legislation.

In contrast, a highlight of this study refers to the possibility of knowing the managers’ point of view, with their limitations, and difficulties and informing them about the resources and government programs to find a win-win path benefiting women, children, and their workplaces. Another relevant point refers to its direct investigation with managers, contributing to scarce results on the subject. The relevance of this study involves its identification of gaps in compliance with maternity protection laws due to managers’ lack of awareness of their responsibilities and the creation of a pro-breastfeeding culture within companies to benefit women, children, and productivity. The concept of the caregiver archetype, which solely assigns women the responsibility of breastfeeding, is a significant contribution of this study, justifying the need for shared responsibility from managers and organizations. Our behavior as individuals suffers the effect of the systems and structures of the environment and society in which we live. Also, social, economic, and political factors influence our knowledge, attitudes, and ability to make healthy choices ^29^.

## Conclusion

The managers’ point of view on breastfeeding and maternity leave duration mostly permeates their knowledge and compliance with the duration of that laborers’ right following labor legislation. Other federal programs and actions the Brazilian government has created more than 10 years ago remain unknown to participants. A company should know and practice at least what is required by law. The findings suggest the need to raise awareness in managers toward an ethical commitment to promoting breastfeeding and preventing discrimination in the workplace.

Many managers apply the knowledge they gained by personal experience to meet the needs of working mothers. We know that this will often fail to suffice given the plurality of scenarios and perceptions laden with beliefs. Thus, we propose greater investments in training actions by healthcare providers or specialists in the field to inform and reinforce investments in existing initiatives, such as the Brazilian Citizen Company Program and the MTA program and to assist the implementation of these strategies, strengthening maternity protection policies. Exclusively assigning the responsibility of breastfeeding to women reinforces the caregiver archetype rooted in society, which contributes to gender discrimination in social, family, and especially work relationships.

## Data Availability

The databases used in the study, including extraction codes, analyses, and results, are available in the repository: https://repositorio.ufu.br/handle/123456789/43345.
